# Supplementary material for: Terahertz-field-induced polar charge order in electronic-type dielectrics
Source: Nat Commun. 2021 Feb 11;12:953. doi: 10.1038/s41467-021-20925-x (PMC7878852; doi:10.1038/s41467-021-20925-x)
Supplement: Supplementary file 1 — Supplementary Information [file 41467_2021_20925_MOESM1_ESM.pdf]

Supplementary Information:

Terahertz-field-induced polar charge order in electronic-type dielectrics

H. Yamakawa[1], T. Miyamoto[1], T. Morimoto[1], N. Takamura[1], S. Liang[1], H. Yoshimochi[2], T. Terashige[3], N. Kida[1], M. Suda[4,6], H. M. Yamamoto[4], H. Mori[5], K. Miyagawa[2], K. Kanoda[2], and H. Okamoto[1,3*]

[1]*Department of Advanced Materials Science, University of Tokyo, Chiba 277-8561, Japan*

[2]*Department of Applied Physics, University of Tokyo, Bunkyo-ku 113-8656, Japan*

[3]*AIST-UTokyo Advanced Operand-Measurement Technology Open Innovation Laboratory, National Institute of Advanced Industrial Science and Technology, Chiba 277-8589, Japan*

[4]*Division of Functional Molecular Systems, Research Center of Integrative Molecular Systems (CIMoS), Institute for Molecular Science, Okazaki 444-8585, Japan*

[5]*Institute for Solid State Physics, University of Tokyo, Chiba 277-8581, Japan*

[6]*Present address: Department of Molecular Engineering, Kyoto University, Kyoto 615-8510, Japan*

* Correspondence author. Email: okamotoh@k.u-tokyo.ac.jp

Supplementary Note 1. Evaluation of polarization magnitudes from terahertz-pump SHG-probe measurements in κ-(ET)$_2$Cu[N(CN)$_2$]Cl

In the terahertz-pump SHG-probe measurements, we used α-(ET)$_2$I$_3$ as a reference material. α-(ET)$_2$I$_3$ is a prototypical two-dimensional (2D) organic conductor. Its 2D conducting layers consist of ET molecules similarly to κ-Cl and κ-CN[1,2]. It was established that α-(ET)$_2$I$_3$ exists in the ferroelectric CO phase below 135 K[3,4]. In the following, α-(ET)$_2$I$_3$ is abbreviated as α-I$_3$. We first measured the steady-state SH signals I_{SHG} at 40 K in α-I$_3$ by irradiating with a near-IR optical pulse (0.95 eV) in the reflection configuration. Then, we replaced the sample with κ-Cl or κ-CN and detected the terahertz-field-induced SH signals. In both κ-Cl and κ-CN, no SHG signal was detected in the steady states at all the temperatures.

Before the quantitative evaluation of the polarization magnitudes, it is useful to compare the important parameters for the SHG, the absorption depths of the incident probe light and the SH light, and the coherence lengths of the SHG processes in the reflection configuration. Those parameters are summarized in Table S1. The absorption depths of both the incident probe light (0.95 eV) and the SH light (1.9 eV), which were calculated by the Kramers-Kronig transformation of the polarized reflectivity spectra, are about 1 μm or less in the three compounds, κ-Cl, κ-CN, and α-I$_3$, in common. These lengths are much shorter than the thicknesses of the crystals used in the measurements. In the reflection configuration used in the present study, the coherence lengths of the SHG (l_{c}) can be evaluated from the following formula.

$$l_{\mathrm{c}} = \frac{\lambda_\omega}{4(n_\omega + n_{2\omega})} \tag{S1}$$

Here, n_ω and $n_{2\omega}$ are refractive indexes for the probe light and the SH light,

respectively, and λ_ω is the wavelength of the probe light. l_c is shorter than 0.1 μm in the three compounds in common as listed in Table S1. Note that the coherence length in the SHG in the reflection configuration is much shorter than that in the transmission configuration. On the other hand, the absorption depth of the terahertz pulse was estimated to be about 100 μm at 1 THz in κ-Cl from the terahertz time-domain spectroscopy reported in Supplementary Note 4. In κ-CN, it is slightly shorter than that in κ-Cl (not shown) but much longer than the coherence length of SHG. Thus, the absorption depths of the incident probe lights, the SH lights, and the terahertz pulse are much longer than the coherence lengths of the SHG, so that the SHG intensities are dominated mainly by the coherence lengths. The coherence lengths of the three compounds are almost equal to each other (Table S1). Therefore, we can roughly estimate the polarization magnitudes from the SH intensities.

The SHG in a non-centrosymmetric material is expressed with the second-order nonlinear susceptibility $\chi^{(2)}(-2\omega;\ \omega,\omega)$ as follows[5].

$$P(2\omega) = \varepsilon_0 \chi^{(2)}(-2\omega;\omega,\omega)E(\omega)E(\omega) \tag{S1}$$

$$I_{\mathrm{SHG}}(2\omega) \propto \left[\chi^{(2)}(-2\omega;\omega,\omega)\right]^2 [I(\omega)]^2 \tag{S2}$$

Here, $E(\omega)$ and $I(\omega)$ are an electric field and an intensity of an incident laser pulse, respectively, and ε_0 is the permittivity of vacuum. $P(2\omega)$ is a nonlinear polarization. In the ferroelectric material of α-$\mathrm{I_3}$, $\chi^{(2)}(-2\omega;\omega,\omega)$ is proportional to the ferroelectric polarization $P_{\alpha-\mathrm{I3}}$. The SH-intensity can be expressed by $I_{\mathrm{SHG}}(\alpha - \mathrm{I_3}) = C_1(P_{\alpha-\mathrm{I3}})^2$. C_1 is a proportional constant. The electric-field-induced SHG in a centrosymmetric material is expressed with the third-order nonlinear susceptibility $\chi^{(3)}(-2\omega;\omega,\omega,0)$ as follows[5].

$$P(2\omega) = \varepsilon_0 \chi^{(3)}(-2\omega; \omega, \omega, 0) E(\omega) E(\omega) E(0) \tag{S3}$$

$$\Delta I_{\mathrm{SHG}}(2\omega) \propto \left[\chi^{(3)}(-2\omega; \omega, \omega, 0)\right]^2 [I(\omega)]^2 [E(0)]^2 \tag{S4}$$

Here, $E(0)$ is a quasi-static electric field. The frequency of the terahertz pulse is much smaller than that of the incident light pulse for SHG and of the optical gap, so that we can consider it equal to zero and $E(0) = E_{\mathrm{THz}}(0)$. $\chi^{(3)}(-2\omega; \omega, \omega, 0) E(0)$ is proportional to the terahertz-electric-field-induced polarization ΔP, and the intensity of the electric-field-induced SHG, $\Delta I_{\mathrm{SHG}}(2\omega)$, is proportional to the square of ΔP. Therefore, $\Delta I_{\mathrm{SHG}}(2\omega)$ can be expressed as $\Delta I_{\mathrm{SHG}}(2\omega) = C_2 (\Delta P)^2$. C_2 is also a proportional constant.

On the basis of the framework mentioned above, The ratio of the SH intensity $\frac{\Delta I_{\mathrm{SHG}}(\kappa-\mathrm{Cl})}{I_{\mathrm{SHG}}(\alpha-\mathrm{I}_3)}$ $\left(\frac{\Delta I_{\mathrm{SHG}}(\kappa-\mathrm{CN})}{I_{\mathrm{SHG}}(\alpha-\mathrm{I}_3)}\right)$ is equal to the square of the ratio of the polarization $\frac{C_2(\Delta P_{\kappa-\mathrm{Cl}})^2}{C_1(P_{\alpha-\mathrm{I3}})^2}$ $\left(\frac{C_2(\Delta P_{\kappa-\mathrm{CN}})^2}{C_1(P_{\alpha-\mathrm{I3}})^2}\right)$. Here, $\Delta I_{\mathrm{SHG}}(\kappa-\mathrm{Cl})$ $(\Delta I_{\mathrm{SHG}}(\kappa-\mathrm{CN}))$ and $\Delta P_{\kappa-\mathrm{Cl}}$ $(\Delta P_{\kappa-\mathrm{CN}})$ are the intensity of the SHG and the magnitude of the polarization induced by the terahertz electric field in κ-Cl (κ-CN), respectively The maximum intensity of the SHG signal shown in Fig. 2c (2d) induced by a terahertz electric-field pulse of the amplitude 407 kV/cm in κ-Cl at 40 K (in κ-CN at 50 K) is approximately 1% (0.6%) of the intensity of the steady-state SHG signal in α-$\mathrm{I_3}$. Here, we assume that the values of the proportional constants C_1 and C_2 in α-$\mathrm{I_3}$ and κ-Cl (κ-CN), respectively, are equal. It is reasonable because the magnitude of the CO gap in α-$\mathrm{I_3}$ is comparable to those of the Mott gaps in κ-Cl and κ-CN. From $\frac{\Delta I_{\mathrm{SHG}}(\kappa-\mathrm{Cl})}{I_{\mathrm{SHG}}(\alpha-\mathrm{I}_3)} \sim 0.01$ and $\frac{\Delta I_{\mathrm{SHG}}(\kappa-\mathrm{CN})}{I_{\mathrm{SHG}}(\alpha-\mathrm{I}_3)} \sim 0.006$, we obtain $\frac{\Delta P_{\kappa-\mathrm{Cl}}}{P_{\alpha-\mathrm{I3}}} \sim 0.1$ and $\frac{\Delta P_{\kappa-\mathrm{CN}}}{P_{\alpha-\mathrm{I3}}} \sim 0.08$. Namely, 10% (8%) of the polarization in α-$\mathrm{I_3}$ is generated by a terahertz electric field in κ-Cl (κ-CN).

The previous density-functional-theory (DFT) calculation revealed that the magnitude of the polarization in the ferroelectric CO phase of α-I_3 is approximately $1\ \mu\mathrm{C/cm^2}$ (Refs. 6 and 7). This yields $\Delta P_{\kappa-\mathrm{Cl}} \sim 0.1\ \mu\mathrm{C/cm^2}$.

Table S1. Absorption depths and coherence lengths associated with SHG

	Absorption depth at 0.95 eV (μm)	Absorption depth at 1.9 eV (μm)	Coherence length in the reflection-type SHG (μm)
α-I_3 at 4K	0.91 (E // a)	0.52 (E // b)	0.084
κ-CN at 10 K	1.27 (E // c)	0.91 (E // c)	0.096
κ-Cl at 10 K	0.44 (E // c)	0.44 (E // c)	0.099

Supplementary Note 2. Analyses of steady-state optical spectra and transient reflectivity changes induced by terahertz electric fields in κ-$(ET)_2Cu[N(CN)_2]Cl$

To clarify the changes in the polarized reflectivity (R) spectra induced by the terahertz electric fields (shown in Fig. 3f,g) in κ-Cl, we first analyzed the steady-state polarized reflectivity (R) and optical conductivity (σ) spectra (Fig. 3a,b). These spectra are almost equal to those reported previously[8].

The structure peaked at 0.43 eV and the broader structure around 0.2 eV observed in the σ spectrum is attributed to the intradimer transition (the orange arrow in Fig. 3c) and the interdimer transition (the green arrow in Fig. 3c), respectively[8]. The three sharp peaks observed at 0.109 eV, 0.157 eV and 0.164 eV are assigned to the intramolecular vibration (a_g) modes[8]. Those modes become IR-active via electron intramolecular-vibration (EMV) coupling. Taking those five types of optical absorption bands into account, we assumed that the complex dielectric constant consists of five Lorentz oscillators as expressed below.

$$\varepsilon(\omega) = \varepsilon_\infty + \sum_{j=1}^{5} \frac{L_j}{\omega_j^2 - \omega^2 - i\omega\Gamma_j} \tag{S2}$$

Here, L_j is the parameter related to the oscillator strength. ω_j and Γ_j are the frequency and the damping constant, respectively, of each oscillator. ε_∞ is the high-frequency dielectric constant. Here, we estimate $\varepsilon_\infty \sim 4$. The terms corresponding to $j = 1$ and 2 represent the interdimer and intradimer transitions, respectively. Those corresponding to $j = 3\text{–}5$ represent the intramolecular vibrations. Using eq. (S2), both the R and σ spectra can be approximately reproduced as illustrated by the red lines in Fig. 3a,b. The parameter values used are listed with error bars (one standard deviation) in Table S2.

Next, we analyzed the spectra of reflectivity changes ΔR induced by the terahertz electric fields shown in Fig. 3f,g. In the analyses, we changed only the oscillator strengths and frequencies of two electronic transitions. That is, the fitting parameters are L_j and ω_j for $j = 1$ and 2. In Fig. 3f,g, the broken lines represent the differential reflectivity spectra between the spectra calculated with the new fitting parameters and that calculated with the original fitting parameters; they reproduce well the experimental $\Delta R(0\ \mathrm{ps})$ and $\Delta R(0.5\ \mathrm{ps})$ spectra. The original and new parameters of L_j and ω_j for $j = 1$ and 2 with error bars (one standard deviation) are compared in Table S3.

At $t_\mathrm{d} = 0\ \mathrm{ps}$, the photon energy $\hbar\omega_1$ of the interdimer transition increases from 207.3 meV to 213.4 meV, i.e., by 6.1 meV. Moreover, the oscillator strength of the intradimer transition decreases by 2.9%, and its photon energy $\hbar\omega_2$ increases from 436.0 meV to 437.8 meV, i.e., by 1.8 meV. However, at $t_\mathrm{d} = 0.5\ \mathrm{ps}$, $\hbar\omega_1$ of the interdimer transition decreases from 207.3 meV to 201.6 meV, i.e., by 5.7 meV; moreover, the intradimer transition shifts to the higher energy by 4.0 meV and its oscillator strength

decreases by 5.4% from the original values. The causes of these spectral changes are discussed in the main text.

Table S2. Fitting parameters of Lorentz oscillators in the ground state

	L_j (eV^2)	ω_j (meV)	Γ_j (meV)
$j = 1$	0.910 ±0.01	207.3 ±0.7	380.1 ±2.5
$j = 2$	0.349 ±0.02	436.0 ±0.2	113.0 ±0.1
$j = 3$	0.0019 ±0.0004	108.9 ±0.2	1.6 ±0.1
$j = 4$	0.0072 ±0.0001	157.5 ±0.2	1.4 ±0.1
$j = 5$	0.011 ±0.0008	164.2 ±0.1	1.6 ±0.1

Table S3. Comparison of the original and new parameters of Lorentz oscillators

	L_1 (eV^2)	ω_1 (meV)	ω_1 (cm^{-1})	L_2 (eV^2)	ω_2 (meV)	ω_2 (cm^{-1})
Ground state	0.910 ±0.01	207.3 ±0.7	1672 ±5.6	0.349 ±0.02	436.0 ±0.2	3517 ±1.6
t_d = 0 ps	0.910 ±0.01	213.4 ±1.4	1721 ±11	0.339 ±0.03	437.8 ±1.0	3531 ±8
t_d = 0.5 ps	0.910 ±0.01	201.6 ±1.7	1626 ±13	0.330 ±0.02	440.0 ±1.2	3549 ±9

Finally, we tried to interpret the changes of the two parameters, the transition energy and the oscillator strength, of the two bands.

(a) The blue shift and decrease of the oscillator strength of the intradimer transition at $t_{\mathrm{d}} = 0$ ps

The photon energy of the intradimer transition is mainly determined by the splitting of the bonding- and antibonding-orbital in each dimer as shown in Fig. 3c, which is approximated to $2t_1$ (t_1: intradimer transfer integral) and equal to U_{dimer} in the Mott insulator state (e.g. refs. 9 and 10). When there is a difference of the site energy potential,

2Δ, between two molecules in a dimer, the splitting is increased to $2\sqrt{t_1^2 + \Delta^2}$ and the charge disproportionation occurs (Fig. 3d). Considering the possibility of the charge disproportionation deduced from the SHG signal by the terahertz electric field, it is natural to consider that this blue shift of the intradimer transition at $t_\mathrm{d} = 0$ ps is due to the generation of the difference in the site energy potential by the terahertz electric field. We consider that this difference would originate not only from the electric field itself, which changes directly the site energy potential, but also from the energy gain in the charge-disproportionated state due to the long-range Coulomb interactions between molecules belonging to different dimers. The generation of the difference in the site energy potential causes the decrease of the hybridization of the molecular orbitals of two molecules in each dimer, which should reduce the oscillator strength of the intradimer transition, as observed in the experiment.

(b) The blue shift of the interdimer transition at $t_\mathrm{d} = 0$ ps

The optical transition energy or Mott-gap energy in a half-filled Mott insulator is determined by the balance of the on-site Coulomb repulsion energy U and the width δ of the upper and lower Hubbard bands. It increases with increase of U and with decrease of δ. In a dimer Mott insulator studied here, U_dimer is approximated to $2t_1$ and δ is determined by the interdimer transfer integrals t and t'. When a difference of the site energy potential, 2Δ, is induced in each dimer, the interdimer transition energy should increase since U_dimer increases from $2t_1$ to $2\sqrt{t_1^2 + \Delta^2}$. Assuming that δ is unchanged, the interdimer transition energy would increase by the terahertz electric field as observed in the experiment.

The explanation of the reflectivity changes $\Delta R(t_{\mathrm{d}} = 0.5\ \mathrm{ps})$ (Fig. 3g) is not so straightforward. Taking into account the interpretation of $\Delta R(t_{\mathrm{d}} = 0\ \mathrm{ps})$ shown above, we can explain the results of $\Delta R(t_{\mathrm{d}} = 0.5\ \mathrm{ps})$.

(c) The blue shift and decrease of the oscillator strength of the intradimer transition at $t_{\mathrm{d}} = 0.5\ \mathrm{ps}$

The delayed further blue shift of the intradimer transition at $t_{\mathrm{d}} = 0.5\ \mathrm{ps}$ suggests that some structural changes would be involved in the dynamics. A possible structural change is the decrease of the dimerization or equivalently the increase in the intermolecular distance between two molecules forming a dimer. It is because the terahertz electric field should not only induce intradimer charge transfer and the resultant charge-disproportionation, but also pull apart two charged molecules in each dimer, which would decrease the dimerization. The time scale of the delayed change is evaluated to be about 0.3 ps from the analysis of the time evolutions of ΔR reported in Supplementary Note 3, which is almost in agreement with the quarter of the period of the breathing mode of dimer. The previous theoretical studies indicate that the reduction of the dimerization, that is, the decrease of the intradimer transfer integral t_1 would enhance the instability to the CO and tend to increase the charge disproportionation in each dimer. In this case, the energy difference, 2Δ, between two molecules in each dimer might be increased possibly via the enhancement of the long-range Coulomb interactions between two molecules belonging to different dimers. In the experiment, the intramolecular transition energy approximated to $2\sqrt{t_1^2 + \Delta^2}$ increases at $t_{\mathrm{d}} = 0.5\ \mathrm{ps}$. This result shows that the increase of 2Δ overcomes the decrease of t_1. The increase of 2Δ and also the decrease of t_1 should suppress the hybridization of π-orbitals between two molecules in each

dimer, reducing the oscillator strength of the intradimer transition, which can explain the experimental result.

(d) The red shift of the interdimer transition at $t_\mathrm{d} = 0.5$ ps

As discussed above, the decrease of the dimerization increases the intradimer transition energy approximated to $2\sqrt{t_1^2 + \Delta^2}$. Since $2\sqrt{t_1^2 + \Delta^2}$ corresponds to U_dimer, this change should increase the intermolecular transition energy. However, the experimental result shows that the interdimer transition energy decreases at $t_\mathrm{d} = 0.5$ ps. To explain this red shift of the interdimer transition, we should consider another factor. A possible factor is the change of the bandwidth; molecular displacements corresponding to the release of dimerization might increase a transfer integral between two molecules belonging to the neighboring dimers. If those transfer integrals increase, the bandwidth would increase, resulting in the decrease of the interdimer transition energy as observed in the experiment. To demonstrate this interpretation, further theoretical studies about the electron-phonon interactions associated with the intermolecular transfer integrals should be necessary.

Supplementary Note 3. Comparison of electric-field-induced SHG and reflectivity changes

In Fig. S1a, we show the comparison of the time evolutions of the electric-field-induced SHG, $\Delta I_\mathrm{SHG}(t_\mathrm{d})$ (Fig. 2c) and reflectivity changes, $-\Delta R(t_\mathrm{d})/R$ (Fig. 5d) for $E_\mathrm{THz}//c$ and $E//c$ in κ-Cl. The photon energies of the incident probe pulses are 0.95 eV and 0.5 eV for the SHG and reflectivity measurements, respectively. The time characteristics of $\Delta I_\mathrm{SHG}(t_\mathrm{d})$ and $-\Delta R(t_\mathrm{d})/R$ are almost the same with each other.

More specifically, the electric field waveforms used in the two measurements are slightly different; the electric-field amplitude of the negative peak around 0.4 ps just after the positive peak is larger in the SHG measurement than in the reflectivity one. However, no difference is observed around 0.4 ps in the time evolutions of $\Delta I_{\mathrm{SHG}}(t_{\mathrm{d}})$ and $-\Delta R(t_{\mathrm{d}})/R$. This suggests that the polarization direction of the polar CO state is dominated by the charge disproportionated state initially generated by the first positive electric-field component around the time origin and that the polarization of the polar CO state is not affected so much by the negative electric field around 0.4 ps.

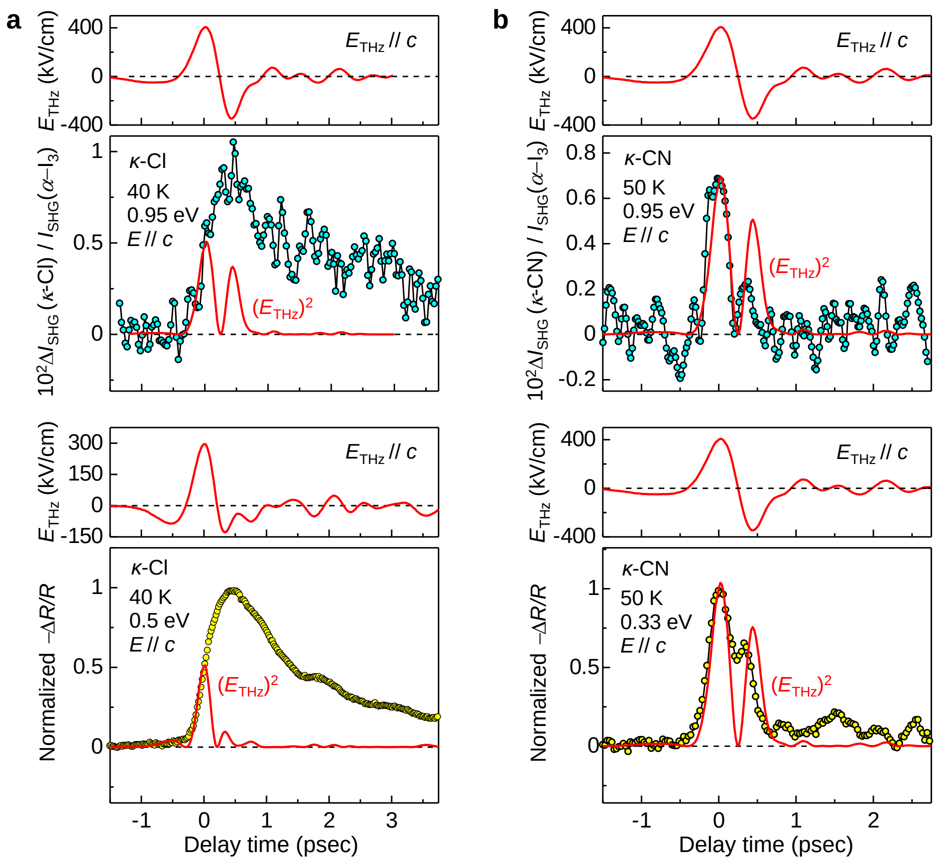

Fig. S1 Comparisons of the time evolutions of electric-field-induced SHG and reflectivity changes at 40 K in *κ*-Cl and in *κ*-CN. **a** The time evolutions of SHG, $\Delta I_{\mathrm{SHG}}(t_{\mathrm{d}})$ (Fig. 2c) and reflectivity changes $-\Delta R(t_{\mathrm{d}})/R$ (Fig. 5d) in *κ*-Cl. **b** The time evolutions of $\Delta I_{\mathrm{SHG}}(t_{\mathrm{d}})$ (Fig. 2d) and $-\Delta R(t_{\mathrm{d}})/R$ in *κ*-CN. The polarizations of pump and probe pulses are parallel to the *c* axis.

In Fig. S1b, we show the similar comparison of the time evolutions of $\Delta I_{\mathrm{SHG}}(t_{\mathrm{d}})$ (Fig. 2d) and reflectivity changes, $-\Delta R(t_{\mathrm{d}})/R$ for $E_{\mathrm{THz}}//c$ and $E//c$ in κ-CN. The photon energies of the incident probe pulses are 0.95 eV and 0.33 eV for the SHG and reflectivity measurements, respectively. The same terahertz pulse was used in those two measurements. The time characteristics of $\Delta I_{\mathrm{SHG}}(t_{\mathrm{d}})$ and $-\Delta R(t_{\mathrm{d}})/R$ are also almost the same with each other, and proportional to the square of the terahertz electric field $[E_{\mathrm{THz}}(t_{\mathrm{d}})]^2$.

We also measured the electric-field-induced SHG, $\Delta I_{\mathrm{SHG}}(t_{\mathrm{d}})$, at 10 K and compared the time evolutions of $\Delta I_{\mathrm{SHG}}(t_{\mathrm{d}})$ and the reflectivity change, $-\Delta R(t_{\mathrm{d}})/R$. In the SHG measurements, we used the terahertz pulse with the higher amplitude ($E_{\mathrm{THz}}(0)$ ~ 700 kV/cm) and the lower incident photon energy (0.65 eV) to improve the signal to noise ratio, and performed the measurements at 10 K and 40 K in the same experimental condition.

In Fig. S2a, we show the time evolutions of $\Delta I_{\mathrm{SHG}}(t_{\mathrm{d}})$ measured with $E_{\mathrm{THz}}(0)$ ~ 700 kV/cm at 40 K by the deep-blue triangles. For comparison, we show the time evolution of $\Delta I_{\mathrm{SHG}}(t_{\mathrm{d}})$ (blue circles) and $-\Delta R(t_{\mathrm{d}})/R$ (yellow circles) shown in Fig. 2c and Fig. 5d, respectively. All the data are normalized at their maximum values. Time characteristics of three data in Fig. S2a are almost the same with each other. In Fig. S2b, we show by the red triangles the time evolution of $I_{\mathrm{SHG}}(t_{\mathrm{d}})$ measured with $E_{\mathrm{THz}}(0)$ ~ 700 kV/cm at 10 K, together with the time evolution of $-\Delta R(t_{\mathrm{d}})/R$ (yellow circles) shown in Fig. 6e. In contrast to the results at 40 K, the time evolutions of $\Delta I_{\mathrm{SHG}}(t_{\mathrm{d}})$ and $-\Delta R(t_{\mathrm{d}})/R$ are considerably different from each other at 10 K. This is attributable to the different nature of the ground states at 10 K and 40 K; the system consists of

disordered or fluctuated microscopic CO domains at 10 K, while it is in the homogeneous Mott insulator state at 40 K.

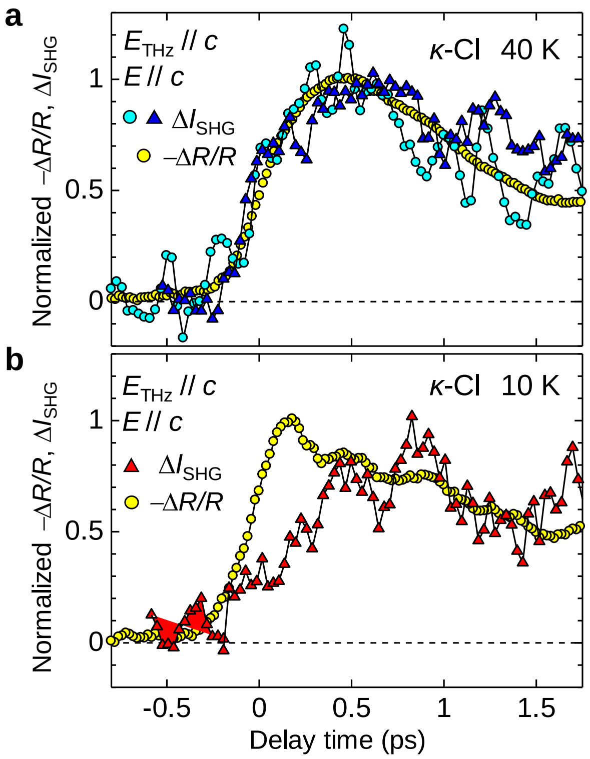

Fig. S2. Comparisons of the time evolutions of electric-field-induced SHG and reflectivity changes at 40 K and 10 K in κ-Cl. **a** Deep-blue triangles show the time evolution of SHG, $\Delta I_{\mathrm{SHG}}(t_{\mathrm{d}})$, measured for the incident (detected) photon energy of 0.65 eV (1.3 eV) with $E_{\mathrm{THz}}(0)$ ~ 700 kV/cm at 40 K. The polarizations of pump and probe pulses are parallel to the *c* axis. Blue and yellow circles show the time evolution of $\Delta I_{\mathrm{SHG}}(t_{\mathrm{d}})$ shown in Fig. 2c and that of $-\Delta R(t_{\mathrm{d}})/R$ shown in Fig. 5d, respectively. **b** Red triangles show the time evolution of $\Delta I_{\mathrm{SHG}}(t_{\mathrm{d}})$ measured for the incident (detected) photon energy of 0.65 eV (1.3 eV) with $E_{\mathrm{THz}}(0)$ ~ 700 kV/cm at 10 K. Yellow circles show the time evolution of $-\Delta R(t_{\mathrm{d}})/R$ shown in Fig. 6e. All the data are normalized by their maximum values.

Supplementary Note 4. Vibrational spectra of κ-$(ET)_2Cu[N(CN)_2]Cl$ in the terahertz region

In this section, we report the σ spectrum in the terahertz region and exclude the likelihood that an infrared-active phonon is excited directly by the terahertz electric field. Figure S3a shows the σ spectrum in the range of 0.3 to 2 THz for the light electric fields $E//c$ at 10 K; this was measured by the terahertz time-domain spectroscopy method[11]. In the σ spectrum in Fig. S3a, several peaks are observed from 1.2 to 1.9 THz; these are ascribed to the intermolecular oscillations. An oscillatory wave observed below 1.2 THz is attributable to the interference of the lights inside the thin single crystal. The magnitudes of σ at the absorption peaks from 1.2 to 1.9 THz are smaller than 3 S/cm and are over two orders of magnitudes smaller than those of the sharp phonon peaks at 38.4 THz and 40.0 THz and the two broad peaks observed in the mid-infrared region. That is, there is no strong absorption in the terahertz region. The red line in Fig. S3a represents the Fourier power spectrum of the electric-field waveform of the pump pulse, whose central frequency is ~ 0.7 THz. From these facts, we can consider that no strong phonon peak is directly excited by the terahertz pulse. Figure S3b shows the Fourier power spectrum of the oscillatory component in $\Delta R(t_{\mathrm{d}})/R$ (process γ). At this oscillation frequency (29 cm^{-1}), no photon absorptions exist. These results demonstrate that the oscillation in $\Delta R(t_{\mathrm{d}})/R$ is not originally infrared-active. As mentioned in the main text, the electric field induces the charge disproportionation in each dimer; this causes the dimer mode to be infrared-active within the duration of the terahertz electric field. As a result, the dimer-mode oscillation is impelled by the terahertz electric field.

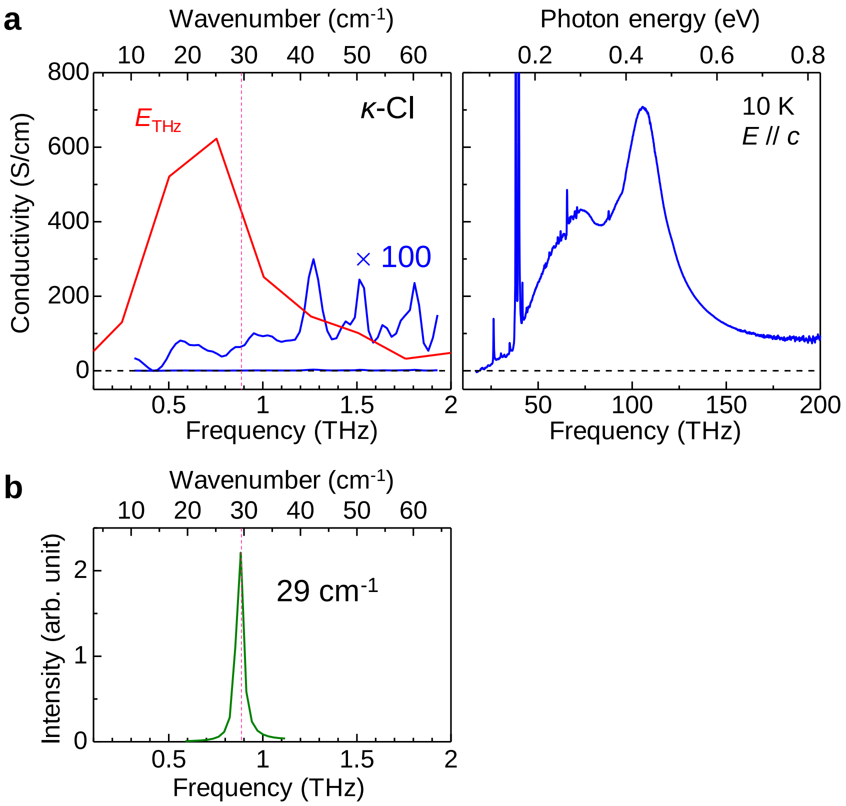

Fig. S3 Spectra of the optical conductivity and the coherent oscillation in κ-Cl. **a** The left panel shows the optical conductivity (σ) spectrum in the range of 0.3-2 THz at 10 K (the blue line) together with the Fourier power spectrum of the terahertz electric-field waveform (the red line). The right panel shows the optical conductivity spectrum in the range of 0.06-0.8 eV. The electric field is parallel to the c axis. **b** The Fourier power spectrum of the coherent oscillation observed in the time evolutions of $\Delta R(t_{\mathrm{d}})/R$ at 0.5 eV and at 40 K ($E_{\mathrm{THz}}(0) = 288\mathrm{kV/cm}$, $E_{\mathrm{THz}}//c$ and $E//c$). The central frequency is ~ 29 cm^{-1}.

Supplementary Note 5. Intermolecular transfer integrals in κ-(ET)$_2$Cu[N(CN)$_2$]Cl and κ-(ET)$_2$Cu$_2$(CN)$_3$

In this section, we summarize the magnitudes of the intermolecular transfer integrals previously reported[12], which would affect both the CO and antiferromagnetic order. Figures S4a and b show the values of the intermolecular transfer integrals $t_i (i = 1\text{–}4)$

in the two-dimensional ET layers in κ-Cl and κ-CN, respectively. t_1 is the intradimer transfer energy, which is almost identical (~200 meV) in the two compounds. In contrast, there is a significant difference in the relative magnitudes of $t_2 - t_4$, between the two compounds: $t_2(102\ \text{meV}) > t_3(67\ \text{meV}) > t_4(43\ \text{meV})$ in κ-Cl and $t_3(91\ \text{meV}) > t_2(85\ \text{meV}) > t_4(17\ \text{meV})$ in κ-CN.

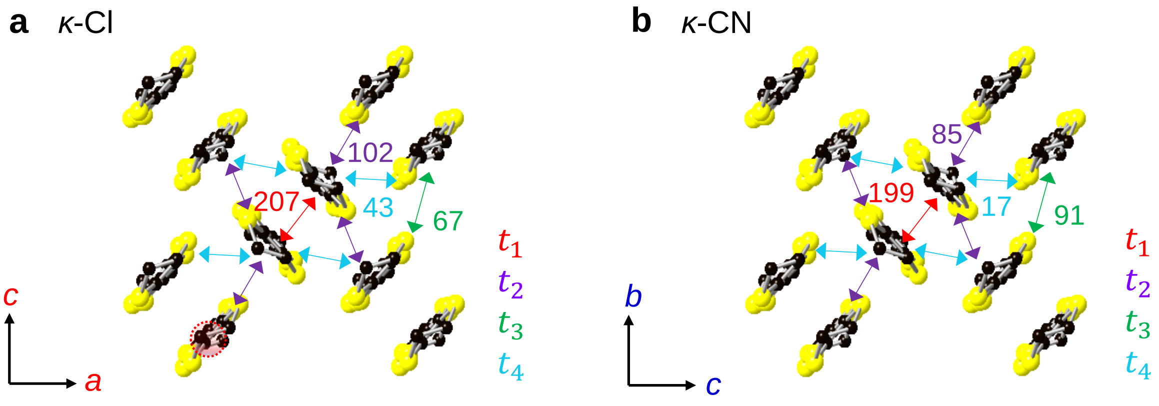

Fig. S4 Comparison of intermolecular transfer integrals in κ-Cl and κ-CN. **a** κ-Cl and **b** κ-CN. All the values of transfer integrals are shown in the unit of meV. The molecular arrangements are schematically shown in both figures.

References

1. Bender, K., Dietz, K., Endres, H., Helberg, H. W., Hennig, I. Keller, H. J., Schäfer, H. W. & Schweitzer, D. $(BEDT\text{-}TTF)^{+}{}_{2}I^{-}{}_{3}$: A two-dimensional organic metal. *Mol. Cryst. Liq. Cryst.* **107**, 45-53 (1984).

2. Bender, K., Hennig, I., Schweitzer, D., Dietz, K. Endres, H. & Keller, H. J. Synthesis, structure and physical properties of a two-dimensional organic metal, di[bis(ethylenedithiolo)tetrathiofulvalene]triiodide, $(BEDT\text{-}TTF)^{+}{}_{2}I^{-}{}_{3}$. *Mol. Cryst. Liq. Cryst.* **108**, 359-371 (1984).

3. Yamamoto, K. *et al*. Strong optical nonlinearity and its ultrafast response associated with electron ferroelectricity in an organic conductor. *J. Phys. Soc. Jpn.* **77**, 074709-074714 (2008).

4. Lunkenheimer, P. *et al.* Ferroelectric properties of charge-ordered α-$(BEDT\text{-}TTF)_2I_3$. *Phys. Rev. B* **91**, 245132 (2015).

5. Butcher, P. N. & Cotter, D. The Elements of Nonlinear Optics (Cambridge University Press, Cambridge, 1990).

6. Yamakawa, H. *et al*. Novel electronic ferroelectricity in an organic charge-order insulator investigated with terahertz-pump optical-probe spectroscopy. *Sci. Rep*. **6**, 20571 (2016).

7. Kuniki, S., Ohmura, S. & Takahashi, A. Charge and dielectric response to terahertz pulses in the charge-ordered phase of α-$(BEDT\text{-}TTF)_2I_3$. *Phys. Rev. B* **98**, 165149 (2018).

8. Faltermeier, D. *et al*. Bandwidth-controlled Mott transition in κ-$(BEDT\text{-}TTF)_2Cu$ $[N(CN)_2]Br_xCl_{1-x}$: optical studies of localized charge excitations. *Phys. Rev. B* **76**, 165113 (2007).

9. Kanoda, K. Metal-insulator transition in *κ*-$(ET)_2X$ and $(DCNQI)_2M$: two contrasting manifestation of electron correlation. *J. Phys. Soc. Jpn.* **75,** 051007 (2006).

10. Faltermeier, D. et al. Bandwidth-controlled Mott transition in *κ*-(BEDT-TTF)$_2$Cu [N(CN)$_2$]Br$_x$Cl$_{1-x}$: optical studies of localized charge excitations. *Phys. Rev. B* **76,** 165113 (2007).

11. Takeda, R., Kida, N., Sotome, M., Matsui, Y. & Okamoto, H. Circularly polarized narrowband terahertz radiation from a eulytite oxide by a pair of femtosecond laser pulses. *Phys. Rev. A* **89**, 033832 (2014).

12. Koretsune, T. & Hotta, C. Evaluating model parameters of the *κ*- and *β*'-type Mott insulating organic solids. *Phys. Rev. B* **89**, 045102 (2014).
